# Induction of apoptosis by directing oncogenic Bcr-Abl into the nucleus

**DOI:** 10.18632/oncotarget.1339

**Published:** 2013-10-09

**Authors:** Zheng-Lan Huang, Miao Gao, Qian-Yin Li, Kun Tao, Qing Xiao, Wei-Xi Cao, Wen-Li Feng

**Affiliations:** ^1^ Department of Clinical Hematology, Key Laboratory of Laboratory Medical Diagnostics Designated by the Ministry of Education, Chongqing Medical University, Chongqing, People's Republic of China; ^2^ Department of Hematology, The First Affiliated Hospital, Chongqing Medical University, Chongqing, People's Republic of China

**Keywords:** chronic myeloid leukemia, Bcr-Abl, nuclear localization, rapalog, apoptosis

## Abstract

The chimeric Bcr-Abl oncoprotein, which causes chronic myeloid leukemia, mainly localizes in the cytoplasm, and loses its ability to transform cells after moving into the nucleus. Here we report a new strategy to convert Bcr-Abl to be an apoptotic inducer by altering its subcellular localization. We show that a rapalog nuclear transport system (RNTS) containing six nuclear localization signals directs Bcr-Abl into the nucleus and that nuclear entrapped Bcr-Abl induces apoptosis and inhibits proliferation of CML cells by activating p73 and shutting down cytoplasmic oncogenic signals mediated by Bcr-Abl. Coupling cytoplasmic depletion with nuclear entrapment of Bcr-Abl synergistically enhances the inhibitory effect of nuclear Bcr-Abl on its oncogenicity in mice. These results provide evidence that direction of cytoplasmic Bcr-Abl to the nucleus offers an alternative CML therapy.

## INTRODUCTION

Chronic myeloid leukemia (CML) is a clonal disease derived from hematopoietic stem/progenitor cells in which Philadelphia (Ph) chromosome forms due to the reciprocal translocation between chromosomes 9 and 22, resulting in the formation of Bcr-Abl oncogene that encodes a constitutively active tyrosine kinase [1-3]. As a non-receptor tyrosine kinase, bcr-abl activates a number of downstream signal transduction pathways participating in the regulation of cell proliferation and apoptosis, including PI3-kinase, Akt, Erk and Stat5 [4,5]. Imatinib mesylate and some second generation kinase inhibitors such as dasatinib and nilotinib are the first choice for CML treatment and have been effective in controlling the disease [6-8]. However, occurrence of drug resistance calls for the development of alternative strategies [9].

Similar to c-Abl protein, Bcr-Abl contains three nuclear localization signals (NLS) and one nuclear export signal (NES). Bcr-Abl mainly localizes in the cytoplasm, whereas c-Abl shuttles between the nucleus and cytoplasm [10]. When stimulated by DNA damage, the nuclear c-Abl kinase is activated to induce expression of p73, a member of the p53 tumor-suppressor family. The cooperation of c-Abl with p73 has been shown to be associated with DNA damage-induced apoptosis [11-13]. Vigneri and Wang [14] have previously shown that nuclear entrapment of Bcr-Abl with active tyrosine kinase activity triggers apoptosis, which was achieved by treatment with imatinib and leptomycin B (LMB) that blocks nuclear export of Bcr-Abl. After the tyrosine kinase activity of Bcr-Abl was recovered by removal of imatinib, the cells underwent spontaneous apoptosis, indicating that entrapment of Bcr-Abl in the nucleus induces apoptosis. This result suggests that the tyrosine kinase activity is required for nuclear Bcr-Abl to induce apoptosis. However, the therapeutic application of LMB is limited by its neuronal toxicity. Dixon et al [15] tested whether ectopically expressed Bcr-Abl could cause apoptosis of K562 cells after being directed to the nucleus via a strong NLS. To do so, they added a single or four NLSs to Bcr-Abl (1NLS-Bcr-Abl or 4NLS-Bcr-Abl) and transfected K562 cells. The result shows that 4NLS-Bcr-Abl translocated to the nucleus and induced apoptosis, whereas 1NLS-Bcr-Abl localized in cell cytoplasm and had no obvious effect on cell apoptosis. Together, these results demonstrate that altering the sublocation of ectopically expressed Bcr-Abl induces apoptosis of CML cells and that multiple NLSs are required to drive Bcr-Abl into the nucleus and induce apoptosis. These results suggest that coupling cytoplasmic depletion with nuclear entrapment of Bcr-Abl may have a synergistic effect on apoptotic regulation of the cells.

In this study, we develop a strategy to direct Bcr-Abl from the cytoplasm into the nucleus by induction of protein heterodimerization of FK506 binding protein (FKBP) and FKBP-rapamycin binding domain (FRB) via AP21967 [16-18]. FKBP is abundant in cytoplasm and serves as the target for rapamycin. Rapamycin functions by binding with high affinity to FKBP, and then to the FRB, thereby acting as a heterodimerizer to facilitate the binding of the two proteins [19]. To use rapamycin for inducing heterodimers between proteins of interest, one of the two proteins is fused to FKBP, and the other to FRB, allowing sufficient binding to form the FKBP-rapamycin-FRB complex. Because rapamycin is an immunosuppressive reagent, chemically modified derivatives of rapamycin with non-immunosuppressive function have been engineered. These compounds, which are called rapalogs, can no longer bind to endogenous FRB, but can still bind to a modified FRB that contains a single mutation (T2098L). Incorporation of this change into the FRB allows a rapalog to specifically heterodimerize with engineered proteins without interfering with the endogenous FRB. AP21967 is one type of the rapalogs and can be used to induce heterodimerization of FKBP and FRB_T2098L_-containing fusion proteins. AP21967 is greater than 1000-fold less immunosuppressive than rapamycin [20-22]. In our study, we designed a strategy called rapalog nuclear transport system (RNTS), by which NLSs were transferred to Bcr-Abl, and as a result, Bcr-Abl was transported into the nucleus. In this study we examined whether RNTS directs Bcr-Abl into the nucleus and depletes it from the cytoplasm, whether RNTS induces apoptosis of CML cells, and the underlying mechanisms. We also evaluated the effect of RNTS on Bcr-Abl oncogenicity in vivo.

## RESULTS

### RNTS directs Bcr-Abl from cytoplasm into nucleus

**Figure 1:**
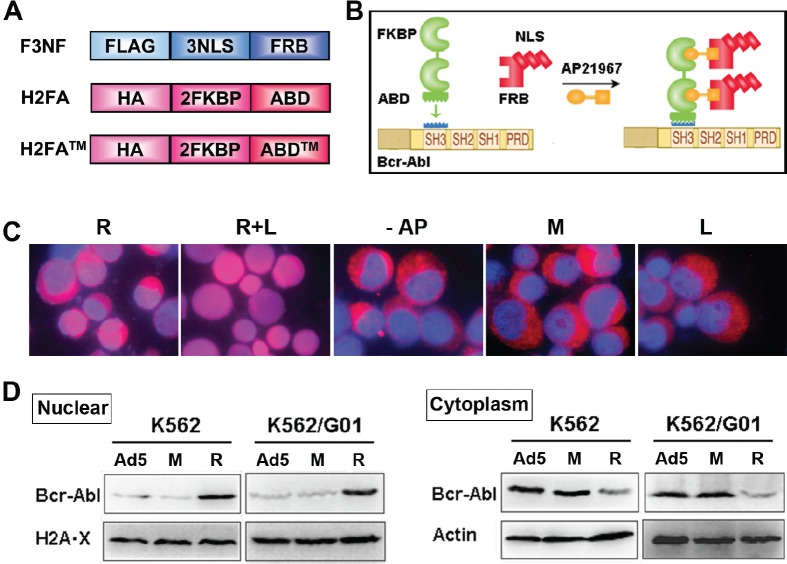
RNTS directs Bcr-Abl from cytoplasm into the nucleus

Three NLSs were fused to FRB_T2098L_ with FLAG tag incorporated to the N-terminus of the protein, which is termed F3NF. Two FKBP domains were in tandem and fused to ABD (Abl binding domain of RIN1) with HA tag fused to the N-terminus, which is termed H2FA (Figure 1A). The ABD of RIN1 interacts with both the SH3 and SH2 domains of Bcr-Abl specifically with high affinity. Because all three potential tyrosine phosphorylation sites Y36, Y121, and Y148 at ABD N-terminus are critical for ABD binding to Bcr-Abl, all three sites were mutated to phenylalanine as a triple mutant, which is termed ABD^™[23]^. Heterodimerization was induced between F3NF and H2FA upon AP21967. Nuclear location signal was transferred from F3NF to H2FA, then to Bcr-Abl (Figure 1B). This strategy is called rapalog nuclear transport system (RNTS). As expected, recombinant F3NF localized mainly in the nucleus, and both H2FA and H2FA™ localized in the cytoplasm (Supplemental Figure 1). In K562 cells, Bcr-Abl located mostly in the cytoplasm. When RNTS was introduced, Bcr-Abl was transported into the nucleus significantly. To retain Bcr-Abl in the nucleus, LMB was added, because LMB is a potent and specific nuclear export inhibitor which functions by inhibiting CRM1/exportin 1, a protein required for nuclear export of NES-containing proteins[24]. When both RNTS and LMB were used, Bcr-Abl was mostly transported into the nucleus (Figure 1C). The amount of Bcr-Abl in the nucleus and cytoplasm was quantified by western blot separately. We found that the level of nuclear Bcr-Abl increased with decreased Bcr-Abl in the cytoplasm (Figure 1D). These results demonstrate that RNTS can direct Bcr-Abl from the cytoplasm and into the nucleus.

(A) Schematic illustration of recombinant proteins F3NF, H2FA and H2FA™. (B) The schematic diagram of RNTS mediated transportation of Bcr-Abl into the nucleus. This figure was derived from modification of the AP21967 instruction picture of Clotech. (C) Nucleocytoplasmic transport was investigated in K562 cells by immunofluorescent assay. Images were merged for two colors: DNA(blue) and Bcr-Abl (red). R: RNTS, L: LMB, AP: AP21967, M: RNTS™, Ad5: Ad5 adenovirus vector. These abbreviations will be used in the rest of the figures. (D) Nuclear and cytoplasmic proteins were collected separately. The amount of Bcr-Abl in the nucleus and cytoplasm was quantified by western blot.

### RNTS binds Bcr-Abl directly

**Figure 2:**
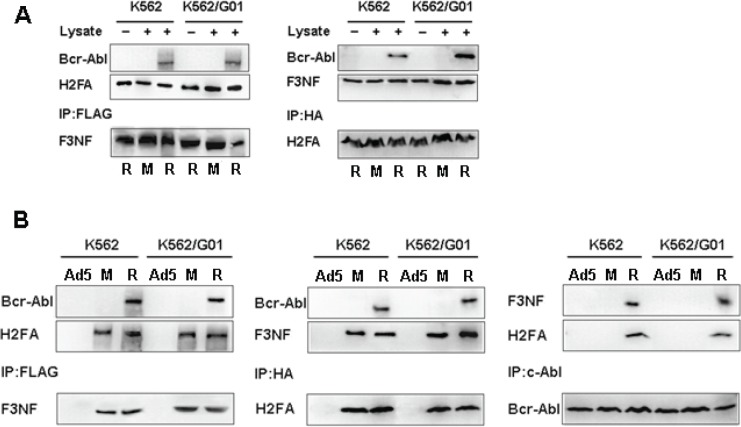
RNTS binds Bcr-Abl directly

We performed pull-down and co-immunoprecipitation assays to examine whether RNTS binds Bcr-Abl directly or indirectly. In the pull-down assay, F3NF, H2FA and H2FA™ were expressed in *E.coli* under IPTG induction, and the His tag contained in the recombinant proteins facilitated protein purification with Ni^+^-NTA. Purified F3NF, H2FA and AP21967 consisted of RNTS in vitro. Bcr-Abl was obtained from K562 and K562/G01 cells. We found that Bcr-Abl was captured extracellularly after immunoprecipitation with either anti-FLAG or anti-HA antibody as detected by western blotting with anti-c-Abl antibody (Figure 2A). We next examined if RNTS could bind to Bcr-Abl intracellularly. K562 and K562/G01 cells were infected with Ad-F3NF and Ad-H2FA (or Ad-H2FA™), and then AP21967 was added to the cell culture medium. Co-immunoprecipitation assay was carried out to detect the interaction between RNTS and Bcr-Abl. When F3NF was captured with anti-FLAG, Bcr-Abl and H2FA were also co-precipitated as detected by western blotting with the corresponding antibodies. Similarly, when anti-HA was used to capture H2FA, Bcr-Abl and F3NF were co-precipitated. Furthermore, when c-Abl antibody was employed to capture Bcr-Abl, F3NF and H2FA were co-precipitated (Figure 2B). Together, these results demonstrate that RNTS binds Bcr-Abl directly.

(A) F3NF, H2FA and H2FA™ are expressed in E.coli and purified with Ni^+^-NTA. Purified proteins and AP21967 consist of RNTS extracellularly. Bcr-Abl protein lysates were collected from K562 and K562/G01 cells. Pull-down assay was performed to detect the interaction between RNTS and Bcr-Abl in vitro. (B) K562 and K562/G01 cells were infected by Ad-F3NF and Ad-H2FA (or Ad-H2FA™), and then AP21967 was added. Co-immunoprecipitation assay was used to detect the interaction between RNTS and Bcr-Abl intracellularly.

### RNTS induces apoptosis and inhibits proliferation of CML cells

**Figure 3:**
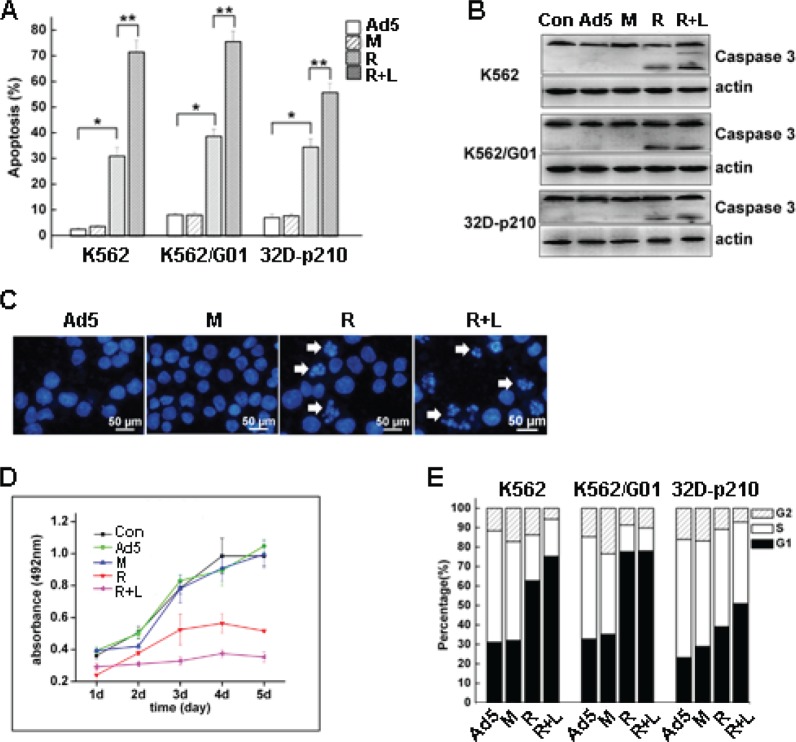
RNTS induces apoptosis and inhibits proliferation of CML cells

To detect the apoptotic effect of RNTS on CML cells, cell surface expression of phosphatidylserine (PS), which is activated by apoptosis, was detected by flow cytometry. Cells treated with RNTS showed a significantly increased apoptotic rate compared to Ad5 vector and mutant control (Figure 3A). RNTS in combination with LMB treatment also significantly increased apoptosis of the cells. In addition, the activated caspase 3, which is a mid-stage apoptosis indicator, was determined by western blot, and we found that cleavage of caspase 3 was merely detected in the cells treated with RNTS alone or in combination with LMB (Figure 3B). Because DNA segmentation is regarded as the most accurate indicator of cell apoptosis, we stained the cells with DAPI and found that nuclear morphology of RNTS treated cells changed from normal round/oval shape to smaller, non-homogeneous segments (Figure 3C). The DNA segmentation was also observed in cells treated with both RNTS and LMB.

K562, K562/G01 and 32D-p210 cells were, respectively, treated with Ad5, RNTS™, RNTS or RNTS in combination with LMB. Apoptosis was detected by flow cytometry (A), cleavage of caspase 3 was detected by western blot (B), and DNA segmentation was detected by DAPI stain (C). The effect of RNTS on CML cell proliferation was assessed by MTS assay (D), and cell-cycle was analyzed by flow cytometry (E).

To assess the effect of RNTS on CML cell proliferation, MTS, colony-forming assay and cell-cycle analysis were conducted. RNTS significantly suppressed CML cell proliferation, and LMB further enhanced the inhibitory effect of RNTS on CML cells (Figure 3D). RNTS also suppressed the colony-forming ability of CML cells (Supplemental Figure 2). Furthermore, flow cytometry analysis showed that RNTS caused a blockade of cell cycle progression from G1 to S phase, especially in K562 and K562/G01 cells (Figure 3E), but LMB only slightly enhanced the cell-cycle blockade induced by RNTS (Figure 3E).

### RNTS activates p73 and its downstream target molecules

**Figure 4:**
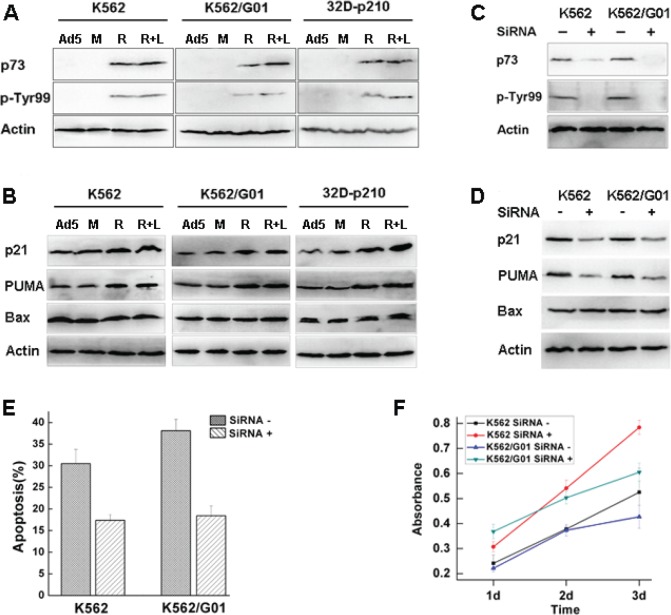
RNTS activates p73 and its downstream target molecules

Nuclear c-Abl kinase can be activated by DNA damage to induce expression of p73 protein, a functional homolog of the tumor suppressor p53, and then p73 induces apoptosis of cells[13]. Therefore, we tested if apoptosis induced by nuclear entrapment of Bcr-Abl caused by RNTS was resulted from p73 activation. We found that the mRNA expression of p73 was upregulated by RNTS treatment (Supplemental Figure 3), and the level of p73 protein was also increased (Figure 4A). Because c-Abl stabilizes p73 by phosphorylation of Tyr99[25], we tested the level of Tyr99 phosphorylation. We found that Tyr99 phosphorylation of p73 was enhanced by RNTS (Figure 4A). Although activation of p73 was associated with increased expression of p21 and PUMA (Supplemental Figure 4, Figure 4B)[26,27], expression of Bax at both mRNA and protein levels was not influenced by p73 activation (Supplemental Figure 4, Figure 4B). It has been shown that p73 does not regulate Bax expression at a transcriptional level[28], and that by interacting with Bax, p73 promotes Bax activation as well as its insertion into the mitochondrial membrane[26]. Thus, in RNTS-treated cells, p73 may regulate Bax function post-transcriptionally. We further tested if the effect elicited by RNTS could be mediated by interacting with p73. p73 was silencing by siRNA, and maximal inhibitory effect was reached at 48 h after transfection (Supplemental Figure 5). Also, the levels of p73 and its phosphorylation were largely reduced by p73 silencing in K562 and K562/G01 cells (Figure 4C). Consistent with the effect on p73, expression of p21 and PUMA were also reduced (Figure 4D). As expected, expression of Bax was not affected (Figure 4D). Apoptosis and growth inhibition induced by RNTS were also reversed by p73 silencing (Figure 4E, F). Taken together, these results provide evidence that RNTS functions by activating p73 and its downstream target molecules.

K562, K562/G01 and 32D-p210 cells were, respectively, treated with Ad5, RNTS™, RNTS or RNTS in combination with LMB. p73 protein and its Tyr99 phosphorylation were tested by western blot (A). The protein expression of p21, PUMA and Bax were analyzed by western blot (B). The effects of p73 siRNA on p73 (C), p21, PUMA and Bax activation (D), apoptotic induction (E), and growth inhibition (F) by RNTS were analyzed.

### Effect of RNTS on the cytoplasmic signaling pathways downstream of Bcr-Abl kinase

**Figure 5:**
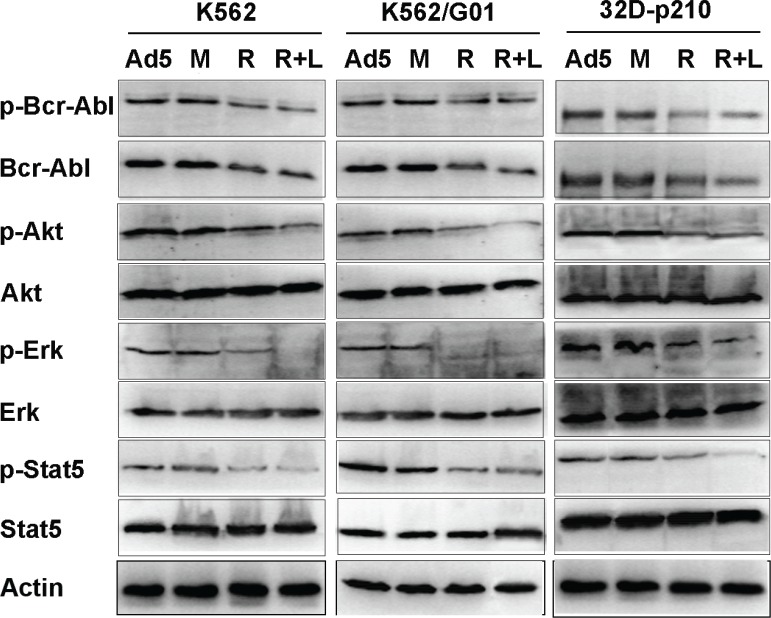
Effect of RNTS on cytoplasmic signaling pathways downstream of Bcr-Abl kinase

Bcr-Abl activates multiple downstream pathways which promote cell proliferation and survival, and suppress apoptosis. Binding of Grb2 SH2 domain to Tyr177 of Bcr-Abl activates Gab2 and Ras, resulting in constitutive activation of PI3K/Akt and Erk[29,30]. Akt activity is essential for Bcr-Abl mediated leukemogenesis and required for both proliferation and survival of Bcr-Abl-expressing cells[31]. Bcr-Abl induces tyrosine phosphorylation and transcriptional activation of Stat5, and the activation of Stat5 plays a role in proliferation and survival of CML cells[29]. Because RNTS directs Bcr-Abl into the nucleus (Figure 1C, D), we reasoned that Bcr-Abl signaling should be compromised. We showed that Bcr-Abl phosphoralytion was reduced, accompanied with decreased expression and phosphorylation of downstream Akt, Erk, and Stat5 (Figure 5).

Cells were respectively treated with Ad5, RNTS™, RNTS or RNTS in combination with LMB. The cytoplasmic protein was prepared. Cytoplasmic Bcr-Abl, Akt, Erk, Stat5 and their corresponding phosphorylation were assessed by western blot.

### RNTS suppresses Bcr-Abl oncogenicity in mice

**Figure 6:**
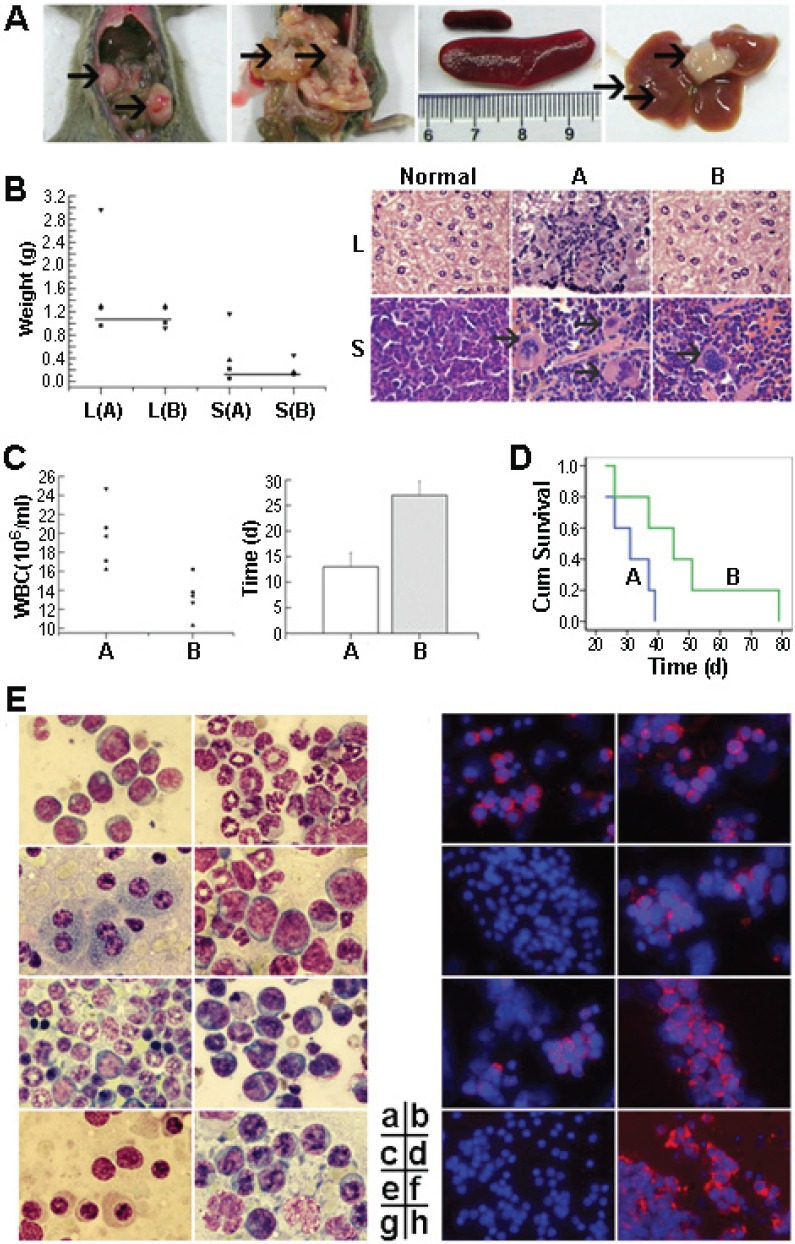
RNTS suppresses Bcr-Abl oncogenicity in mice

Our in vitro studies described above show that RNTS depleted cytoplasmic Bcr-Abl by directing it into the nucleus, leading to induction of apoptosis and inhibition of proliferation of CML cells. To increase the significance of these findings, we tested if RNTS inhibits tumorigenesis of 32D-p210 cells in vivo. 32D-p210 cells treated without or with RNTS were injected into C3H mice intravenously. Mice injected with untreated or RNTS treated 32D-p210 cells were termed group A or B, respectively. Mice began to develop leukemic symptoms starting around three weeks after injection of tumor cells, including weight loss, hind-limb paralysis, reduced activity, and fluffy hair. Pathologically, we observed splenomegaly in almost all morbid mice (Figure 6A), with more severe splenomegaly in group A than in group B (Figure 6B). In detail, RNTS treatment significantly reduced splenomegaly in 32D-p210 transplanted mice (P < 0.05) (spleen weight was 0.45 ± 0.49 g for group A and 0.22 ± 0.16 g for group B). Hepatomegaly and liver tumor nodule was observed in some mice (liver weight of group A: 1.62 ± 0.91 g; group B: 1.13 ± 0.19 g). Three mice from group A had solid tumor in enterocoelia (among which one mouse had two tumors), and only one mouse from group B had enterocoelia tumor. Two mice from group A had mesenteric infiltration (Figure 6A). By HE staining, we found that spleen samples from group A displayed severe infiltration of myeloid cells. In contrast, samples from group B showed little myeloid infiltration (Figure 6B). Similar pathological changes were observed in the liver (Figure 6B). Blood samples were collected every five days after transplantation to determine the level of leukocytes. Morbid mice had increased leukocytes detected by Wright's staining of peripheral blood smears (supplemental Figure 6). Compared to group A, mice in group B had significantly fewer leukocytes (P < 0.05) [group B: (13.0 ± 2.7) × 10^6^ cells/ml, n = 5; group A: (19.7 ± 3.4) × 10^6^ cells/ml, n = 5] (Figure 6C). In addition, RNTS treatment significantly prolonged the time for reaching the highest WBC count (P < 0.05) (group A: 13.0 ± 2.7 d; group B: 27.0 ± 2.7 d) (Figure 6C). Consistently, RNTS treatment significantly prolonged disease latency (31.2 ± 6.9 days for group A and 47.6 ± 19.9 days for group B; P < 0.05). The experimental mice all died of CML-like disease. The difference in survival of the diseased mice was compared, and Kaplan-Meier curves showed that RNTS significantly prolonged survival of the mice (P < 0.05) (Figure 6D). Finally, we determined tissue infiltration of leukemic cells by Wright's stain and immunofluorescent assay for detecting Bcr-Abl (Figure 6E). We found that Bcr-Abl expression was detected in myeloid cells in the bone marrow and spleens of all morbid mice (Figure 6E-a, b, e, f). In the livers, three mice from group A and one mouse from group B had infiltration of Bcr-Abl-expressing cells, other mice expressed no Bcr-Abl protein (Figure 6E-c, d). Cells from enterocoelia solid tumor expressed a high level of Bcr-Abl and morphology of these cells was consistent with that of immature myeloid cells (Figure 6E-h). In the kidneys, there was no infiltration of Bcr-Abl-expressing cells (Figure 6E-g).

32D-p210 cells untreated (group A) or treated (group B) with RNTS were collected and injected into C3H mice intravenously. (A) Display of enterocoelia tumor, mesenteric infiltration, splenomegaly and tumor nodule in the livers. (B) Comparison of liver and spleen weight (left) and staining of tissue by HE (right). The solid lines indicate the normal mean values. L: liver, S: spleen. (C) Comparison of the highest levels of WBC counts between mice in groups A and B (left). Average times (days) for reaching the highest WBC counts in groups A and B (right). (D) Kaplan-Meier survival curves of mice in groups A and B. (E) Tissue infiltration analysis by Wright's staining and detection of Bcr-Abl protein by immunofluorescent assay. a,b: bone marrow; c,d: liver; e,f: spleen; g: kidney; h: enterocoelia solid tumor.

## DISCUSSION

Imatinib has been the first choice for treating CML patients in chronic phase, and has raised the prospect of CML therapy with high efficacy and fewer side effects [32,33]. However, it does not cure CML. Patients need to take it for the remainder of their lives [34]. Moreover, imatinib has little efficacy on CML patients who progressed into the acute phase [35]. Importantly, due to prolonged exposure to imatinib, CML cells could develop drug resistance due to the development of Bcr-Abl kinase domain point mutations [36]. Hence, alternative therapies are needed for CML patients.

The cytoplasmic Bcr-Abl is a potent inhibitor of apoptosis and accelerator of proliferation. The anti-apoptotic and pro-proliferative activity of Bcr-Abl contribute to the initiation and development of CML [37,38]. In this study, we provide evidence that nuclear translocation of Bcr-Abl can compromise its cell transformation ability and induce apoptosis of CML cells. Under RNTS treatment, a significant portion of Bcr-Abl protein was transported into the nucleus and sufficient to induce apoptosis, which could not be overcome by remaining cytoplasmic Bcr-Abl. Induction of apoptosis of CML cells by nuclear Bcr-Abl is consistent with stimulation of cell death induced by DNA damage [11,39]. Because c-Abl kinase induces p73 and activates its pro-apoptotic function [11], activation of p73 and its downstream target molecules by nuclear Bcr-Abl suggests that nuclear Bcr-Abl induces apoptosis similarly. It is reasonable to think that cytoplasmic depletion of Bcr-Abl compromises its ability to activate downstream signal transduction pathways that promote cell proliferation and transformation. Furthermore, by inhibiting the activity of cyclin-CDK2 or -CDK1 complexes, p21 acts as a regulator of cell-cycle progression at G1 [40]. Stat5 regulates cell-cycle progression from G1 to S phase by upregulating cyclin D1 [41]. It is likely that upregulation of p21 and downregulation of cytoplasmic p-Stat5 by RNTS cause G1 arrest of CML cells, which is reflected by the in vivo synergistic inhibitory effect on Bcr-Abl oncogenesis by nuclear localization and entrapment of Bcr-Abl.

Escort of Bcr-Abl from cytoplasm to the nucleus is a promising therapeutic strategy for converting Bcr-Abl from an oncoprotein to an apoptotic inducer. In this study, we show that Bcr-Abl can be directed into the nucleus by attaching six NLSs to a Bcr-Abl binding domain, and that nuclear entrapment of Bcr-Abl can induce apoptosis and inhibit proliferation of CML cells through activating p73 and depleting cytoplasmic oncogenic signals downstream of Bcr-Abl. The oncogenic suppression by nuclear Bcr-Abl suggests that the ability of RNTS to direct Bcr-Abl to the nucleus has great potential for being used to induce apoptosis of CML cells, even for imatinib resistant leukemia cells and CML stem cells. Most importantly rapamycin and its analogs are also indicated for the treatment of leukemia with minimal side effects [42], including CML, especially drug-resistant cases [43]. So the benefit could be two-fold based on our current study.

Imatinib, dasatinib or nilotinib triggers durable responses in many CML patients. Recently, the multi-resistant T315I mutation, which is insensitive to these TKIs, has been circumvented by ponatinib, which is highly effective on both sensitive and resistant CML cell lines, and also on BaF3 murine B cells carrying native Bcr-Abl or T315I mutation [44]. However, the clinical application of ponatinib remains to be validated. Targeting leukemic stem cells (LSC) is another method to cure CML. Chomel et al [45] have demonstrated the long-term persistence of a considerable amount of Bcr-Abl-expressing stem cells in patients which are in a status of undetectable molecular residual disease, even in the absence of relapse. The phenomenon of long-term LSC dormancy is of major importance in CML, because the most primitive stem cells are refractory to all TKIs. Thence, it is urgent to develop a rational reagent targeting LSC in CML.

We used adenovirus as a delivery vector for RNTS, because adenoviral vectors have been widely used for transient genetic manipulation of malignant cells. Transient and high level expression of a gene carried by an adenoviral vector is beneficial to gene delivery [46]. Although clinical application of adenoviral vectors in patients is not practicable at present, our study proves the principle that RNTS delivered by improved delivery methods will provide a new therapeutic strategy for CML patients.

## MATERIALS AND METHODS

### Cell lines and cell culture

K562, K562/G01 and 32D-p210 cell lines were grown and maintained in RPMI-1640 medium supplemented with 10% fetal bovine serum (Gibco, USA). All cells were cultured and maintained in a 37°C incubator with a humidified atmosphere at 5% CO_2_. K562 cells were cultured in gradually increased concentrations of imatinib for several months to generate the resistance line, termed K562/G01, and no point mutation in the Bcr-Abl ATP-binding site was detected although the copy number of Bcr-Abl fusion gene was increased in K562/G01 cells[47]. The 32D-p210 cell line was generated from 32D cell line transformed by p210^Bcr-Abl^ [48].

### Antibodies

We purchased following antibodies: anti-H2A·X, anti-Bax, anti-p73, anti-Phospho-p73 (Tyr 99), anti-Stat5 (3H7), anti-Phospho-Stat5 (Tyr 694) (C71E5), anti-caspase 3, anti-Abl, anti-Akt, and anti-Phospho-Akt (Ser 473) (Cell Signaling Technology, USA); anti-Erk 1/2 (MK1), anti- Phospho-Erk 1/2 (Thr 202/Tyr 204), anti-p21 (C-19), anti-PUMA α/β (D-20), anti-β-actin, horseradish peroxidase (HRP)-conjugated goat anti-rabbit/mouse immunoglobulin G (IgG) and HRP-conjugated rabbit anti-goat (Santa Cruz Biotechnology, USA); anti-FLAG (Sigma, USA).

### Generation of recombinant adenovirus expressing F3NF, H2FA or H2FA™

FLAG tag and three NLSs were chemically synthesized. FRB was amplified by PCR using human cDNA as a template. F3NF was linked by overlapping PCR, then cloned into pAdTrack-CMV and verified by sequencing. HA was chemically synthesized. FKBP was generated by PCR using human cDNA as a template. ABD and ABD™ were amplified from pGEX-RIN1N or pGEX-RIN1N™, respectively. HA, 2FKBPs and ABD were stepwise cloned into pAdTrack-CMV. The resulting fused sequence was termed H2FA. The construction of H2FA™ was done similarly. The expression cassette was used to generate recombinant adenovirus using the AdEasy system as previously described[49]. Cells were infected with adenoviruses at an MOI of 10^5^:1.

### Immunofluorescence microscopy

Cultured cells were grown on poly-L-lysine coated chamber slides. Cells were fixed in 4% paraformaldehyde, and then incubated in ice-cold acetone. The cells were pre-blocked in 5% goat serum in 1% BSA/0.2% Triton X-100/PBS, and then incubated with the primary antibody in 1% BSA/0.05% Triton X-100/PBS at 4°C overnight. Next, the cells were incubated with fluorochrome-conjugated secondary antibodies in 1% BSA/0.05% Triton X-100/PBS. The nucleus was stained with 1 μg/ml DAPI for 5 min.

### Western blot

Protein lysates were obtained by lysing cell pellets with RAPI lysis buffer. 100 μg of each protein sample was loaded into each well, and transferred onto a PVDF membrane. The membrane was blocked in 5% nonfat milk/TBST, and then incubated with primary antibody overnight. The membrane was washed, then incubated with HRP-conjugated secondary antibody, and developed with enhanced chemiluminescence substrate (ECL) (Millipore, USA). Chemiluminescent bands were visualized on cool image workstation II (Viagene, USA).

### Pull-down assay

F3NF, H2FA and H2FA™ were cloned into pET-32a(+) and expressed in BL21λDE3 (pLysS) under IPTG induction. Proteins were purified with Ni^+^-NTA affinity chromatography resin (Novagen, USA), and then filtered to remove the imidazole. Pierce Classic IP Kit was used to pull down the protein of interest. K562 and K562/G01 cell pellets were lysed with IP Lysis/Wash Buffer. The supernatant was pre-cleared using the Control Agarose Resin. Pre-cleared protein lysates were combined with 1 μg antibody, 15 μg purified F3NF, 15 μg purified H2FA (or H2FA™), and 2μl AP21967 (0.5mmol/L) (purchased for Clotech, also known as A/C heterodimerizer) in 500 μl of IP Lysis/Wash Buffer, and incubated for 6 h at 4°C. 20 μl of Protein A/G Plus Agarose was added to the IP mixture and incubated by gentle end-over-end mixing for 4 h at 4°C. The immune complex was captured by centrifugation, eluted by the Non-reducing Lane Marker Sample Buffer, and then run on a SDS-PAGE gel for western blot analysis.

### Co-immunoprecipitation assay

Protein lysates were added into an Anti-HA spin column (anti-HA agarose), and incubated at 4°C for 4 h with end-over-end mixing. The column was spun for 10 seconds, and wash three times with TBST (pulse centrifuge for 10 seconds after each wash). HA-tagged proteins were eluted by the Elution Buffer, and run on a SDS-PAGE gel for western blot analysis. Immunoprecipitation of F3NF and c-Abl was done as described above in the pull-down assay.

### Apoptotic and cell cycle analysis by flow cytometry

Cells were treated with Ad5, RNTS™, RNTS alone or in combination with LMB, respectively. For apoptotic analysis, cells were stained with AnnexinV-PE and 7-AAD (KeyGenBiotech, China) according to the manufacturer's instructions. For cell cycle analysis, cells were fixed in 70% ethanol, and then incubated in staining solution. Cell cycle phase distribution was monitored by a Beckman/Coulter EPICS Elite flow cytometer. Each experiment was repeated three times.

### MTS assay

Cell growth was analyzed by the MTS assay. 2×10^3^ cells in exponential phase were plated into each well in 96-well plates, with three duplications for each treatment. After incubation, 10 μl of MTS solution (Promega, USA) was added to each well, incubated for 2 h at 37°C, and then the absorbance was measured.

### p73 silencing by siRNA

Cells were plated one day before transfection. p73 siRNA was purchased from Santa Cruz Biotechnology company (USA). The transfection was performed according to the manufacturer's instruction. The final concentration of siRNA was 20 pmol.

### Ethics statement

Investigation has been conducted in accordance with the ethical standards and according to the Declaration of Helsinki and according to national and international guidelines and has been approved by the Chongqing Medical University Animal Care and Use Committee.

### Murine Bcr-Abl leukemogenesis assay

Three-to-four-week-old female C3H mice were used. 2×10^6^ of 32D-p210 cells treated with or without RNTS were injected intravenously into C3H mice as previously described[50] (n=5 for each experimental group). The mice will be monitored for weight loss, failure to thrive, splenomegaly, and hind-limb paralysis. Pre-morbid mice were sacrificed. Hind-limb paralysis was scored based on the ability of a mouse to use hind limbs for ambulation on a countertop.

### Hematoxylin-eosin (HE) staining and Wright's staining

4 μm paraffin sections were cut, dewaxed in xylene, re-hydrated in descending alcohol series, and stained using a routine hematoxylin-eosin staining technique as described previously[51]. Air-dried cell smears were stained with Wright's staining solution A (BaSO, China) for 30 seconds and with the solution B for 45 seconds, and rinsed with tap water for observation.

### Statistical analysis

Results were presented as the mean ± SD. Statistical analysis was performed using Student's t test with P < 0.05 deemed as statistically significant. Kaplan-Meier survival curves were graphed using SPSS 13.0.

## Supplementary Figures


