# Supplementary material for: Induction of apoptosis by directing oncogenic Bcr-Abl into the nucleus
Source: Oncotarget. 2013 Oct 9;4(12):2249–60. doi: 10.18632/oncotarget.1339 (PMC3926824; doi:10.18632/oncotarget.1339)
Supplement: Supplementary file 1 [file oncotarget-04-2249-s001.pdf]

Induction of apoptosis by directing oncogenic Bcr-Abl into the nucleus - Huang et al

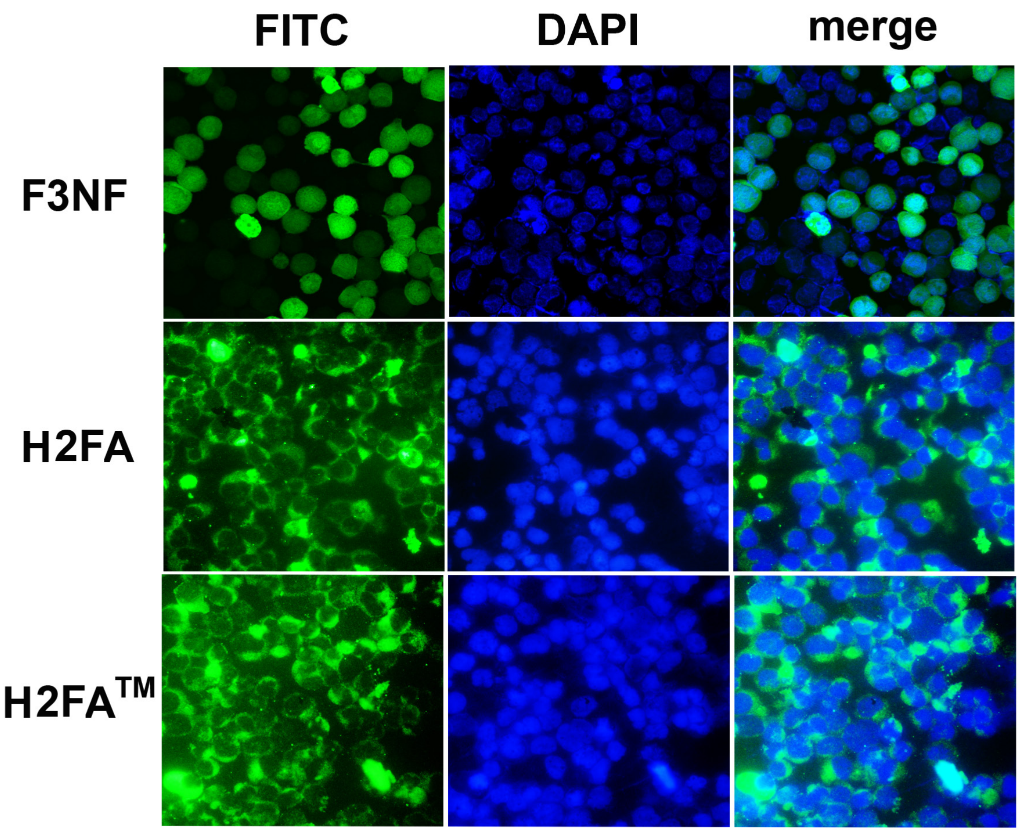

Supplemental figure 1: The subcellular localization of F3NF, H2FA and H2FATM was observed by immunofluorescent assay.

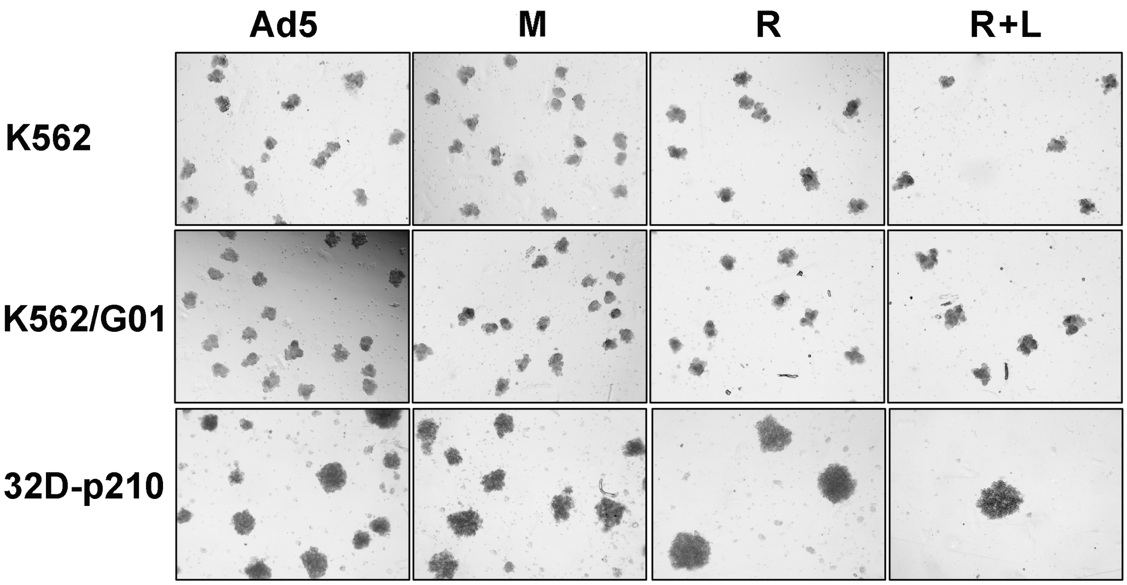

Supplemental figure 2: The result of methylcellulose colony-forming assay.

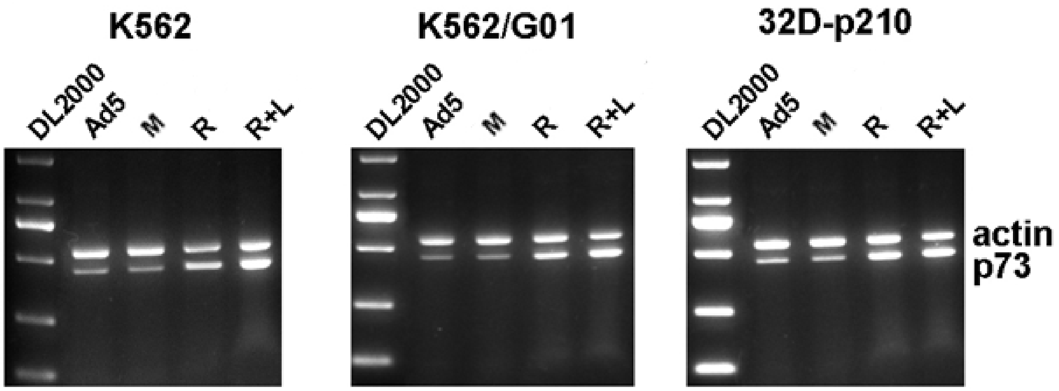

Supplemental figure 3: The mRNA level of p73 was detected by RT-PCR.

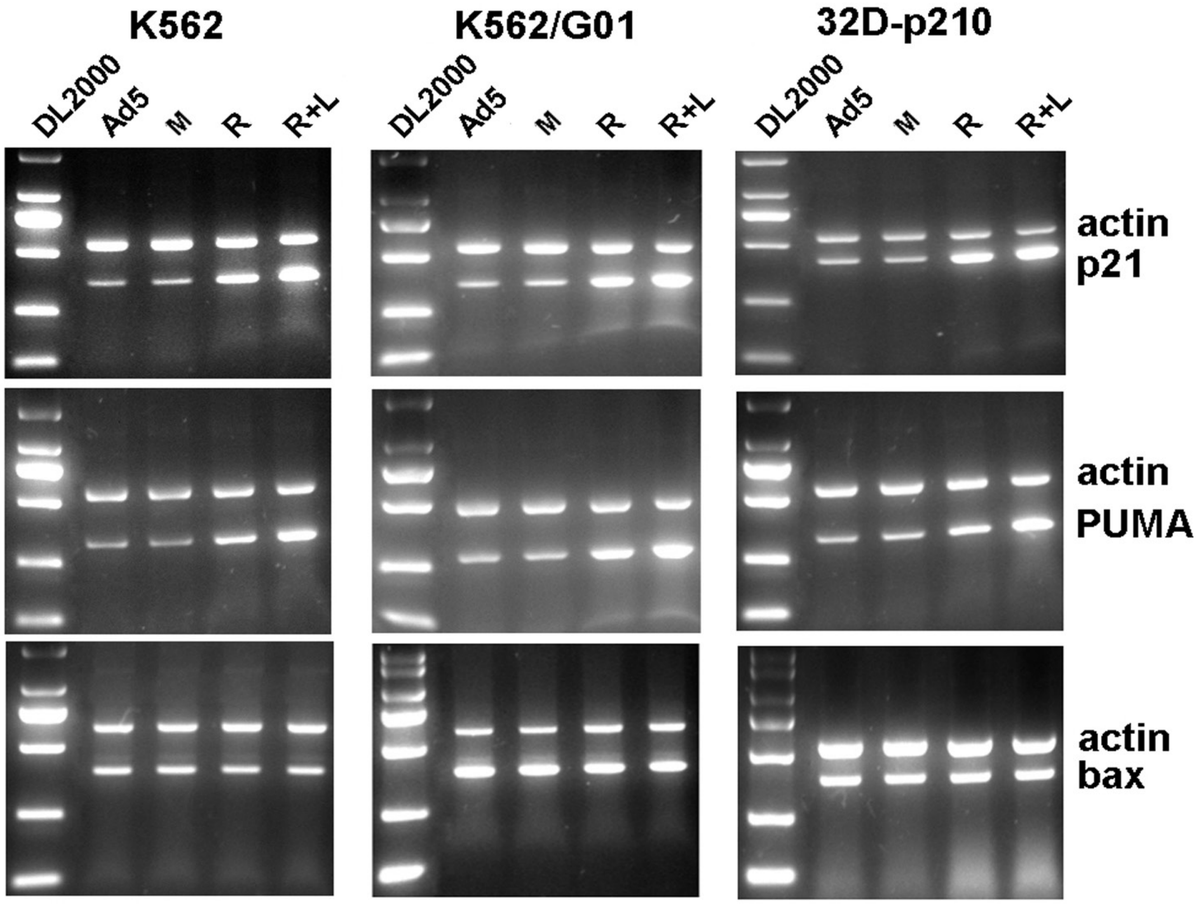

Supplemental figure 4: The mRNA level of p21, PUMA and Bax was analyzed by RT-PCR.

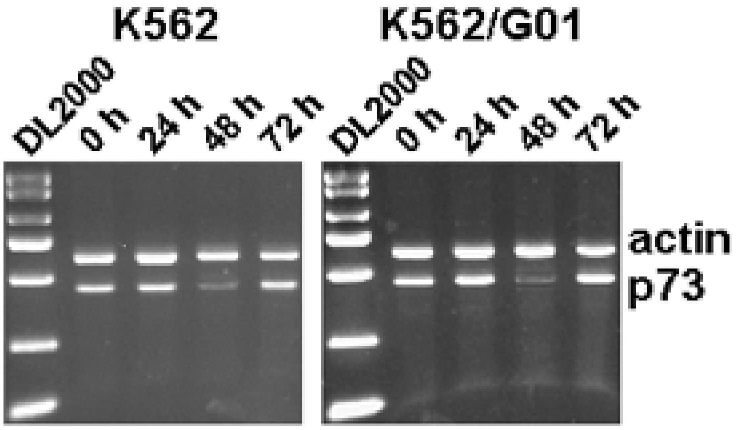

Supplemental figure 5: The maximum inhibitory effect of siRNA on p73 was detected by RT-PCR.

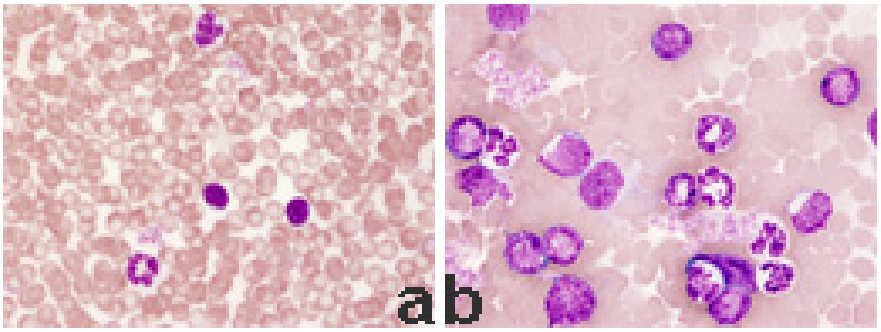

Supplemental figure 6: Wright's staining of peripheral blood smears of mice. (a) normal peripheral blood smear, (b) peripheral blood smear from morbid mouse.
